# Nonlinear Asymmetric Blood Oxygenation Level Dependent Responses in Somatosensory Cortex

**DOI:** 10.1002/hbm.70523

**Published:** 2026-04-07

**Authors:** Feng Wang, Pai‐Feng Yang, Arabinda Mishra, Li Min Chen, John C. Gore

**Affiliations:** ^1^ Vanderbilt University Institute of Imaging Science, Vanderbilt University Medical Center Nashville Tennessee USA; ^2^ Department of Radiology and Radiological Sciences Vanderbilt University Medical Center Nashville Tennessee USA; ^3^ Department of Biomedical Engineering Vanderbilt University Nashville Tennessee USA

**Keywords:** activtion, blood oxygenation level dependent (BOLD), deactivation, fuctional MRI (fMRI), hemodynamic response function (HRF), multi‐unit activity (MUA), nonlinearity

## Abstract

Blood oxygenation level dependent (BOLD) responses in fMRI have previously been shown to be nonlinear with regard to changes in stimulus parameters, and as a result they may be asymmetric when comparing stimulus increases with decreases from an initial condition. We measured BOLD responses to varying vibrotactile stimuli of the hand digits in a monkey, including both increases and decreases in intensity and duration, relative to different levels of initial activation. Across variations in stimulus duration and intensity, positive and negative BOLD responses were asymmetric and nonlinear. Moreover, the asymmetry between positive and negative responses was manifest at different levels of baseline activation. The results confirm that the use of a common hemodynamic response function for increases and decreases in activity may underestimate the magnitude of decreases in activation. Electrophysiological recordings from multi‐electrode arrays also revealed nonlinear and asymmetric features in multi‐unit activities, linking neural firing properties to the nonlinear BOLD profiles.

## Graphical abstract

- BOLD responses to increments and decrements in intensity of vibrotactile stimulation were asymmetric.
- Negative BOLD effects (decrements in signal) were smaller in amplitude and slower to peak compared with positive BOLD responses to increases of intensity.
- BOLD responses were nonlinear for changes in stimulus duration and intensity.

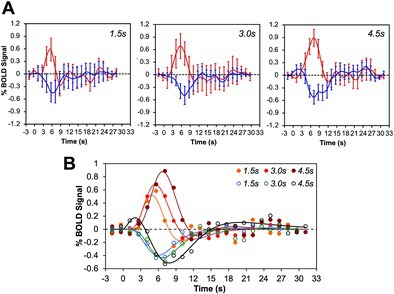

## 1 Introduction

Functional MRI studies of brain activation usually detect signal increases caused by blood oxygenation level dependent (BOLD) effects, which have been shown to be directly coupled to increases in neural activity (Devor et al. 2003; Kayser et al. 2004; Logothetis et al. 2001; Mukamel et al. 2005; Niessing et al. 2005; Sheth et al. 2003; Smith et al. 2002; Thompson et al. 2003) that stimulate concomitant rises in cerebral blood flow (CBF) and cerebral blood volume (CBV). Increases in BOLD signals correspond to reductions in deoxyhemoglobin concentration in tissue, which result in decreases in the transverse relaxation rate R_2_*. In event‐related paradigms, transient increases in neural activity evoked by a stimulus produce a hemodynamic response function (HRF) whose temporal waveform is assumed in most analyses of BOLD signals. The physiological basis of the HRF has been extensively studied since the original balloon model of Buxton et al. (Buxton et al. 1998).

In addition to positive BOLD responses, some fMRI studies also report decreases in MR signal, which we designate as negative BOLD responses or NBRs. In a typical study, NBRs could arise from decreases in neural activity (e.g., inhibition or adaptation effects) induced by a task in different neural networks, which are conventionally regarded as deactivations (Hlushchuk and Hari 2006; Shmuel et al. 2006, 2002). However, in addition, decreases of neural response are expected within a primary target of a task if a continuous stimulation is briefly interrupted. Moreover, given that during a continuous stimulus an equilibrium is reached between flow and oxygenation, and that decreases in BOLD signal represent increases in the concentration of deoxyhemoglobin and/or decreases in blood volume, it is plausible that the degree of BOLD signal change induced by an interruption may depend on the initial level of activation.

Although NBRs have been widely observed, the temporal characteristics and modeling of their corresponding HRFs have been less well documented. Early studies demonstrated that positive BOLD responses (PBRs) exhibit distinct nonlinearities (Birn and Bandettini 2005; Boynton et al. 1996; Liu and Gao 2000; Robson et al. 1998). For example, responses to prolonged stimulation cannot be reliably predicted by linear summation of the responses to short stimuli. While most experimental designs may try to minimize this confound, commonly used general linear model (GLM) analyses implicitly assume linearity.

Another form of nonlinearity is manifest if there are differences in magnitudes between responses to equivalent increases and decreases in neural activity. Most analyses assume that such decreases mirror activations in temporal form and magnitude, modeling them with inverse HRFs (Buxton and Frank 1997; Duysens et al. 1996; Friston et al. 2000; Hyder 2004; Logothetis et al. 2001; Mayhew et al. 2001; Obata et al. 2004; Ogawa et al. 1993). However, Birn and Bandettini (2005) showed that BOLD decreases following short stimulus interruptions were smaller than predicted by linear models, and Tang et al. (Tang et al. 2009) demonstrated that HRFs for deactivations in visual cortex (V1) differ significantly from those for activations. In their work, brief stimulus interruptions evoked NBRs with lower amplitude than the corresponding PBRs, indicating that the HRFs for the two conditions are not reciprocal. These findings show that BOLD responses to increases and decreases in neural activity are different, which will affect the results of studies that assume symmetry.

We extended previous studies by examining NBRs associated with reduced neural activity in the somatosensory cortex and tested the degree to which HRF asymmetry may depend on baseline activity levels. We focused on a single cortical region (area 3b) under a controlled and constant activation state. Using a well‐established squirrel monkey model of activation in primary somatosensory area 3b (Chen et al. 2007; Zhang et al. 2007, 2010), we quantified BOLD responses under varying stimulation conditions, including changes in duration and intensity from both resting and elevated baselines. We first characterized PBRs as a function of vibrotactile stimulus duration and compared them to NBRs elicited by short interruptions of continuous stimulation. We then assessed responses to increases or decreases in stimulus intensity and duration relative to an elevated baseline. Finally, we modeled differences between PBRs and NBRs and compared them with multi‐unit activity (MUA) recorded intra‐cortically at the same sites in subsequent sessions. These electrophysiological data provided direct measures of the neural activity changes underlying the observed BOLD responses.

We emphasize that the NBRs in the stimulated regions caused by stimulus interruption in this work are not equivalent to task‐specific deactivation or suppression in surrounding or non‐stimulated regions widely described in the literature (Hlushchuk and Hari 2006; Shmuel et al. 2006, 2002).

## 2 Materials and Methods

### 2.1 Animal Preparation

Squirrel monkeys (*n* = 2) were sedated with ketamine hydrochloride (10 mg/kg, i.m.) and atropine (0.05 mg/kg, i.m.), intubated, and maintained under isoflurane anesthesia (0.8%–1.1%) in a 30:70 O_2_/N_2_O mixture with mechanical ventilation. Animals were placed in a custom MR cradle with head stabilization using front head and ear bars. Lactated Ringer's solution (2–3 mL/h/kg, i.v.) was infused to prevent dehydration. Physiological parameters, including SpO_2_, heart rate (Nonin, Plymouth, MN), ET‐CO_2_ (22–26 mmHg; Surgivet, Waukesha, WI), ECG, respiration, and temperature (SA Instruments, Stony Brook, NY), were continuously monitored. Body temperature was maintained (37.5°C–38.5°C) using a circulating water blanket (Gaymar Industries, Orchard Park, NY). Real‐time monitoring continued from induction until recovery. All procedures were approved by the Vanderbilt University Institutional Animal Care and Use Committee.

### 2.2 MRI Data Acquisition

MRI data (4 sessions for each animal) were acquired using a 9.4T Varian INOVA scanner (21‐cm bore, Varian Medical Systems, Palo Alto, CA) with a 3‐cm surface transmit‐receive coil positioned over the sensory cortex contralateral to the stimulated hand (Qi et al. 2016). Scout gradient‐echo images were acquired for planning, optimizing field homogeneity, and defining oblique slices covering the primary somatosensory cortex. Functional images were acquired using a GE‐EPI sequence (TR = 1500 ms, TE = 20 ms, 64 × 64 matrix, 0.625 × 0.625 × 2 mm^3^ resolution). Four slices were obtained with a field of view of 40 × 40 × 8 mm^3^. High‐resolution T_2_*‐weighted structural images (TR/TE = 200/16 ms; 4 slices; 512 × 512; 78 × 78 × 2000 μm resolution) were collected to visualize cortical vasculature for localization and registration of fMRI maps.

**FIGURE 1:**
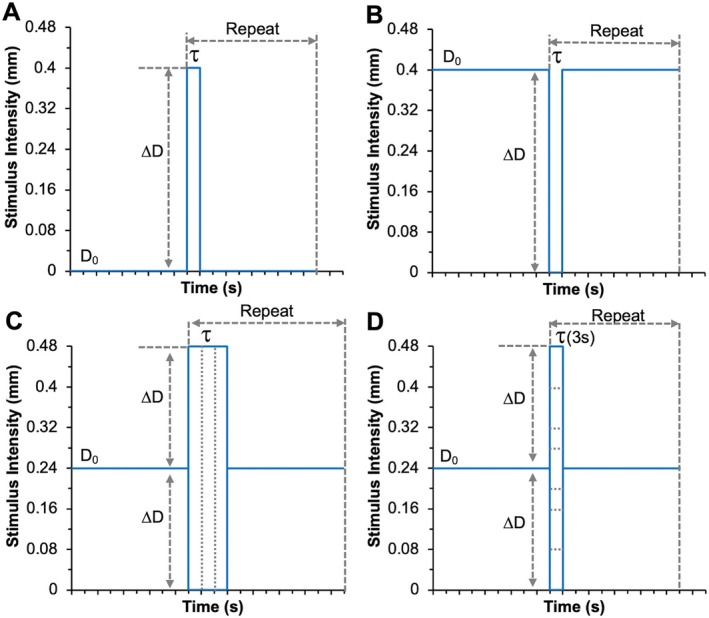
Paradigms of activation and deactivation vibrotactile stimulus presentation. (A, B) Conventional paradigm and the reversed one with the same stimulus intensity (0.40‐mm probe displacement) when the stimulation is on. In paradigm A, conventional stimulation starting from a resting baseline (*D*_0_ = 0 mm) followed by stimulation with consistent intensity but altered duration *τ* (0.5, 1.0, 1.5, 3.0 and 4.5 s). In paradigm B, the reverse paradigm starting from a steady‐state level of stimulation (*D*_0_ = 0.40 mm), interrupted by altered intervals of durations without stimulation. The intervals were 27 s. (C, D) Activation and deactivation starts from the same stimulation baseline (*D*_0_ = 0.24 mm). In paradigm C, the stimulation has consistent intensity change (Δ*D* at ±0.24 mm probe displacement) but altered duration *τ* (1.5, 3.0, and 9.0 s). In paradigm D, the vibrotactile strength is increased with different amplitude in the activation or decrease at the same scale in the deactivation (change of displacement Δ*D* at ±0.04, ±0.08, ±0.16 or ±0.24 mm) with a consistent duration 3 s.

Fingers were immobilized by attaching pegs to the nails and embedding them in modeling clay, leaving finger pads accessible for vibrotactile stimulation by a 2‐mm probe driven by a piezoelectric device (Noliac, Kvistgaard, Denmark). The probe displacement D (0.04–0.48 mm) was proportional to driving voltage (10–120 V) and thus determined stimulation intensity. The probe maintained light skin contact before stimulus onset (*D*_0_ = 0 mm). During each fMRI run, digits D2 and D3 were stimulated simultaneously (vibration frequency 8 Hz, displacement ΔD). We collected 2–4 runs (25 stimulus epochs/runs, 6–15 min) for each condition of the four different event‐related paradigms tested (Figure 1) in each session. These paradigms were:

- Activation from resting baseline: Stimuli (*D*_0_ = 0 mm, Δ*D* = 0.40 mm) of varying duration (0.5–9 s) with 27‐s interstimulus interval.
- Deactivation from steady‐state stimulation (*D*_0_ = 0.40 mm, Δ*D* = −0.40 mm): Interrupted by variable no‐stimulus intervals matched to paradigm A.
- Symmetric intensity changes: From a steady‐state stimulation baseline (*D*_0_ = 0.24 mm), transient increases or decreases (Δ*D* = ±0.24 mm) for different durations (1.5–9 s), 27 s interval.
- Graded intensity changes: From a steady‐state stimulation baseline (*D*_0_ = 0.24 mm), transient 3 s stimulus intensity changes with ΔD from ±0.04 to ±0.24 mm.

A block design (30 s ON and 30 s OFF, 7 stimulus epochs) was also included in each session to confirm robust BOLD responses.

### 2.3 Intracortical Microelectrode MUA Recordings

To map somatotopy and neuronal responsiveness in areas 3b, MUA recordings were performed in the same monkeys following the fMRI experiments, as described previously (Jain et al. 1997; Merzenich et al. 1978; Qi et al. 2011). Briefly, monkeys were sedated with ketamine (10–30 mg/kg, i.m.) and maintained on isoflurane (1%–2% in O_2_) during surgery. After craniotomy, the dura was removed, the exposed cortex was covered with silicone oil, and the cortical surface was photographed to guide electrode placement and align with MRI maps.

To characterize neuronal receptive fields, a tungsten microelectrode (1 MΩ, Microprobe) was inserted perpendicularly with a microhydraulic manipulator (Narishige, NY) at ~300 μm spacing, avoiding blood vessels. Electrode penetrations targeted D2 and D3 representations in area 3b. Neuronal responses were monitored acoustically while advancing to 700 μm depth. For each site, receptive fields were mapped and neurons classified as cutaneous (light touch), high threshold (tapping), or deep (joint/muscle). The minimum receptive field was determined by the area of skin where light touches with a hand‐held probe activated the recorded neurons (Merzenich et al. 1978). Each recording trial included: 1‐s pre‐stimulus, 4‐s baseline vibration (0.24‐mm displacement, 8 Hz), a 3‐s increase or decrease (Δ*D* from ±0.04 to ±0.16 mm), 4‐s baseline vibration, and 1‐s post‐stimulus. Nine trials were recorded per condition.

### 2.4 Functional MRI Data Preprocessing and Detection of Stimulus‐Driven Activations and Deactivations

All fMRI data underwent motion and slice timing corrections (Mishra et al. 2025). The time courses of the EPI data were detrended and temporally smoothed using a low‐pass filter; no spatial smoothing was applied. To model stimulus‐driven BOLD responses, the stimulus paradigm was convolved with a canonical HRF and used as a predictor. Voxel‐wise correlations between the BOLD signal time courses and the stimulus predictor were calculated using a generalized linear model (GLM, spm12). Activation (stimulus increases) or deactivation (stimulus decreases) maps were generated at the false discovery rate corrected threshold of *q* < 0.05. For each condition, BOLD signals were averaged across runs. The fMRI data were realigned and coregistered with high‐resolution T_2_*‐weighted structural images in individual subject space. For visualization, activation and deactivation maps at 64 × 64 resolution were interpolated to 512 × 512 and overlaid on corresponding structural images.

### 2.5 Characteristics of BOLD Response Profiles

The time courses of the signals of the two voxels showing the most significant responses within the region of interest (ROI) were analyzed for both activation and deactivation conditions. The ROI was defined based on activation and deactivation maps and kept consistent across conditions to ensure comparability. The signal was averaged over a 6‐s window preceding stimulus onset and used as the baseline to calculate percentage signal changes. These percentage BOLD signal changes were then averaged across epochs for each stimulus condition within a fMRI run. Subsquently, the averaged responses were further aggregated across runs, sessions, and subjects. To characterize the temporal dynamics of the BOLD responses, a double gamma‐variate function was fitted to the averaged time courses. This model yielded key response parameters, including time to peak (TTP), peak amplitude, and area under the curve. Linearity of the BOLD response was assessed by regression analyses across different stimulus conditions.

### 2.6 MUA Analysis and Correlation With BOLD Characteristics

While a high‐impedance electrode was used to maximize the detection of neural spiking activity, the recorded local field potential (LFP) signals were relatively weak and less robust than MUA. To quantify MUAs, which reflect the combined electrical signals from several nearby neurons, raw neural recordings were high‐pass filtered (cutoff: 300 Hz) to isolate spike activity. Spikes were detected using a threshold‐based method, and counts were computed in 100‐ms bins. Normalization was applied to reduce the effects of differences in stimulation baselines (Figure S1A). Spike counts at each time point were adjusted relative to the sustained stimulation baseline (Figure S1A) before they were averaged across trials. This normalization ensured comparability across trials by aligning baseline levels (Figure S1B). The temporal profile of multi‐unit response was then calculated as:
(1)

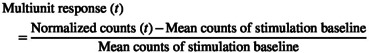




Due to clear differences in multi‐unit responses between early transition and later sustained periods, changes in MUA (ΔMUA) were quantified using peak amplitude and mean values across three time windows: 0–300 (transition), 300–3000 (sustained), and 0–3000 ms (entire response period) during both activation or deactivation conditions. Pearson's correlation coefficients were calculated to assess relationships between BOLD signal characteristics (e.g., peak amplitude, area under the curve) and ΔMUA across varying stimulation intensities.

## 3 Results

### 3.1 Reproducible Focal Positive and Negative BOLD Signals to Brief Tactile Stimuli

**FIGURE 2:**
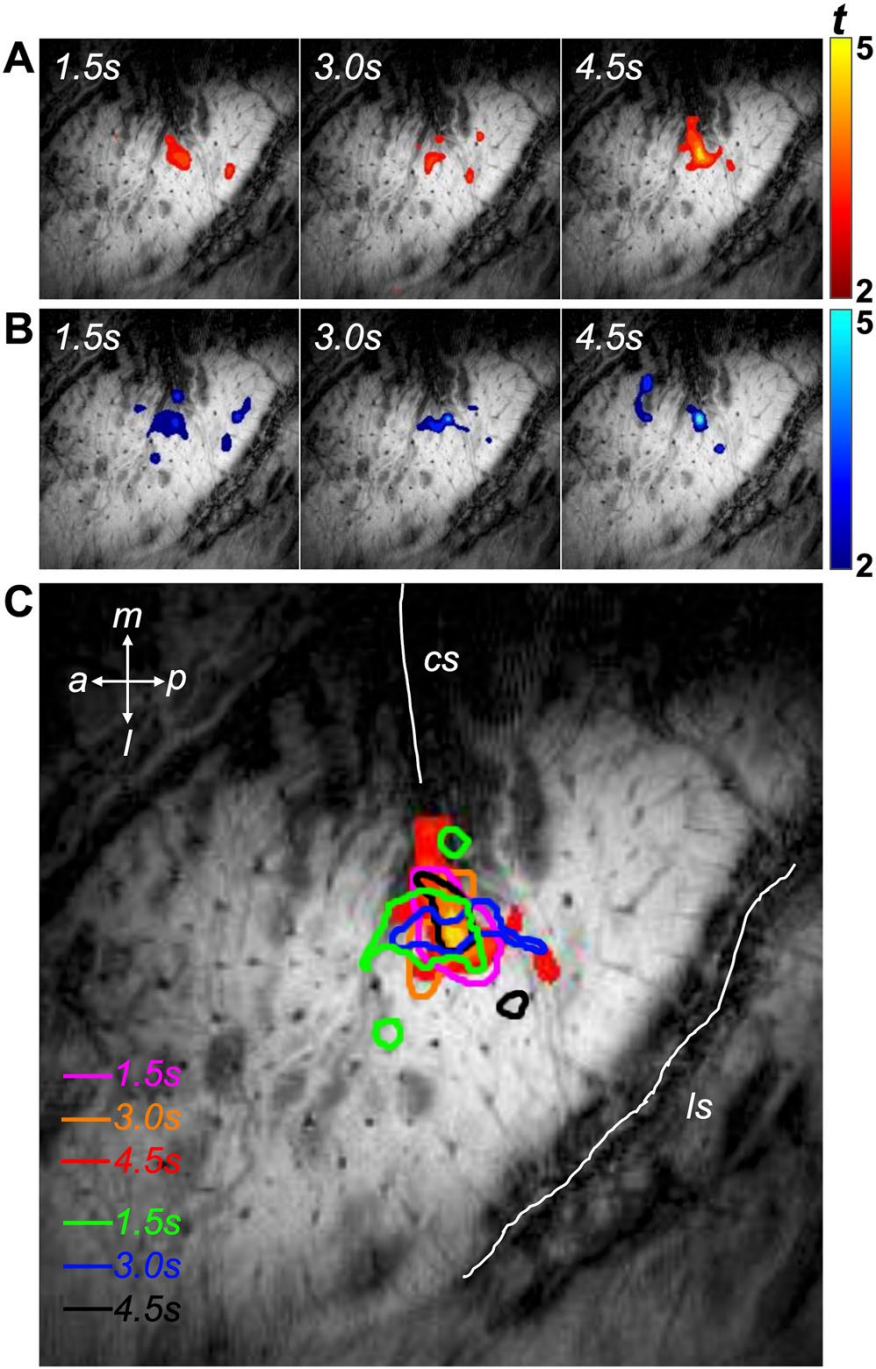
BOLD responses in areas 3b and 1 of primary somatosensory cortex SI. (A) Positive activations for stimulus ON duration at 1.5, 3.0 and 4.5 s (displacement at 0.40 mm) from paradigm A shown in Figure 1. Red for positive BOLD responses. (B) Negative deactivation for interruption with stimulus OFF duration at 1.5, 3.0 and 4.5 s (displacement at 0.40 mm) from paradigm B. Blue for negative BOLD responses. (C) The outlines of the activation and deactivation areas from positive and negative responses shown in 2A‐B. cs: central sulcus; ls: lateral sulcus; m: middle; p: posterior; a: anterior; l: lateral. The averaged responses across runs from one representative subject were shown.

Vibrotactile stimulation using paradigm A (stimulus ON for 1.5, 3.0, and 4.5 s from resting baseline) reliably evoked focal positive BOLD responses in area 3b of primary somatosensory cortex (S1) (Figure 2A). The reverse paradigm B (stimulus OFF for 1.5, 3.0, or 4.5 s during steady‐state stimulation) elicited negative BOLD responses in the same region (Figure 2B). In both cases, activations and deactivations in area 3b were robust and spatially consistent across runs, with smaller, more variable responses in area 1, consistent with previous findings (Chen et al. 2007). To illustrate overlap, composite maps from six stimulation paradigms confirmed that centers of positive and negative BOLD responses coincided within area 3b (Figure 2C). Thus, both increases and decreases in vibrotactile input reliably produced stable, focal BOLD responses in area 3b, providing a model for testing linearity in response to changes in stimulus duration and intensity.

### 3.2 Asymmetric Response Profiles of Positive and Negative BOLD Signals

**FIGURE 3:**
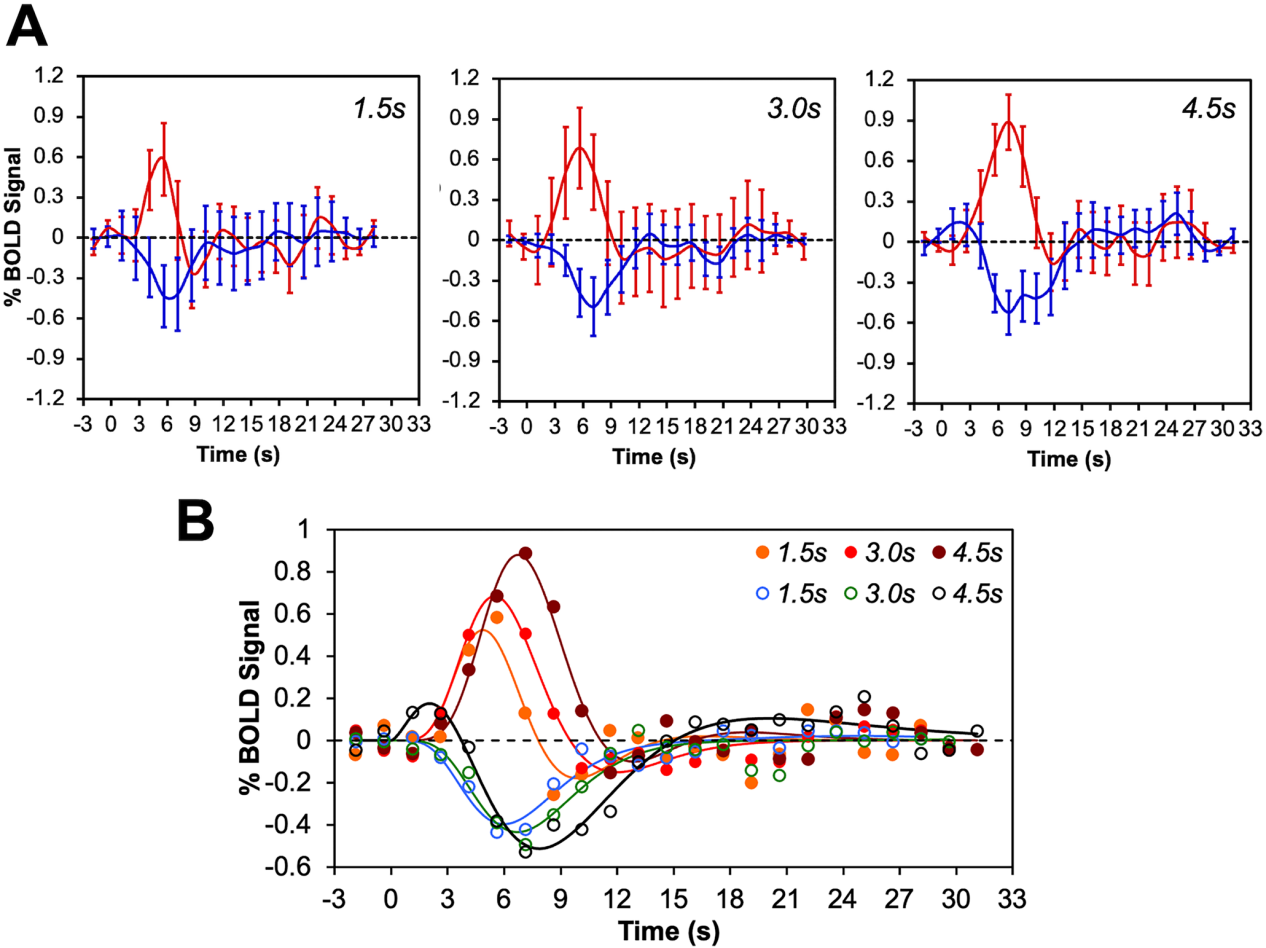
The average event‐related BOLD signal changes in area 3b in response to different stimulus interruption. (A) Measured BOLD responses for stimulus duration 1.5 s, 3.0 s and 4.5 s respectively. Activation in red and deactivation in blue. Error bars show standard deviations across epochs. (B) Fitted BOLD responses, using the gamma variate function. The filled markers for activations and blank markers for deactivations. The *r* ^2^ values are 0.828, 0.932, and 0.919 for brief stimuli of 1.5, 3, and 4.5 s durations respectively (paradigm A in Figure 1). The *r* ^2^ values are 0.928, 0.837 and 0.926 for corresponding stimulation interruptions of 1.5, 3, and 4.5 s durations (paradigm B in Figure 1).

As shown in Figure 3, BOLD responses were well fit by a gamma‐variate function (*r* ^2^ = 0.828–0.932 across conditions). Compared with PBRs to brief stimuli ON, NBRs to stimuli OFF had smaller amplitudes, lacked post‐stimulus overshoots, and rose more slowly (longer TTP). The observation of asymmetric PBRs and NBRs to the same stimulus amplitude changes (increase or decrease) is similar to previous observations in human visual cortex (Birn and Bandettini 2005; Tang et al. 2009). However, one observation that was not reported in human V1 cortex is the slower signal rising slope for NBR in area 3b of somatosensory cortex, which is reflected by longer TTP for NBR compared to PBR (Figure 3). In some experiments, we also examined the BOLD signals to even shorter stimuli of 0.5 and 1.0 s. The response profiles are shown in Figure S2.

**FIGURE 4:**
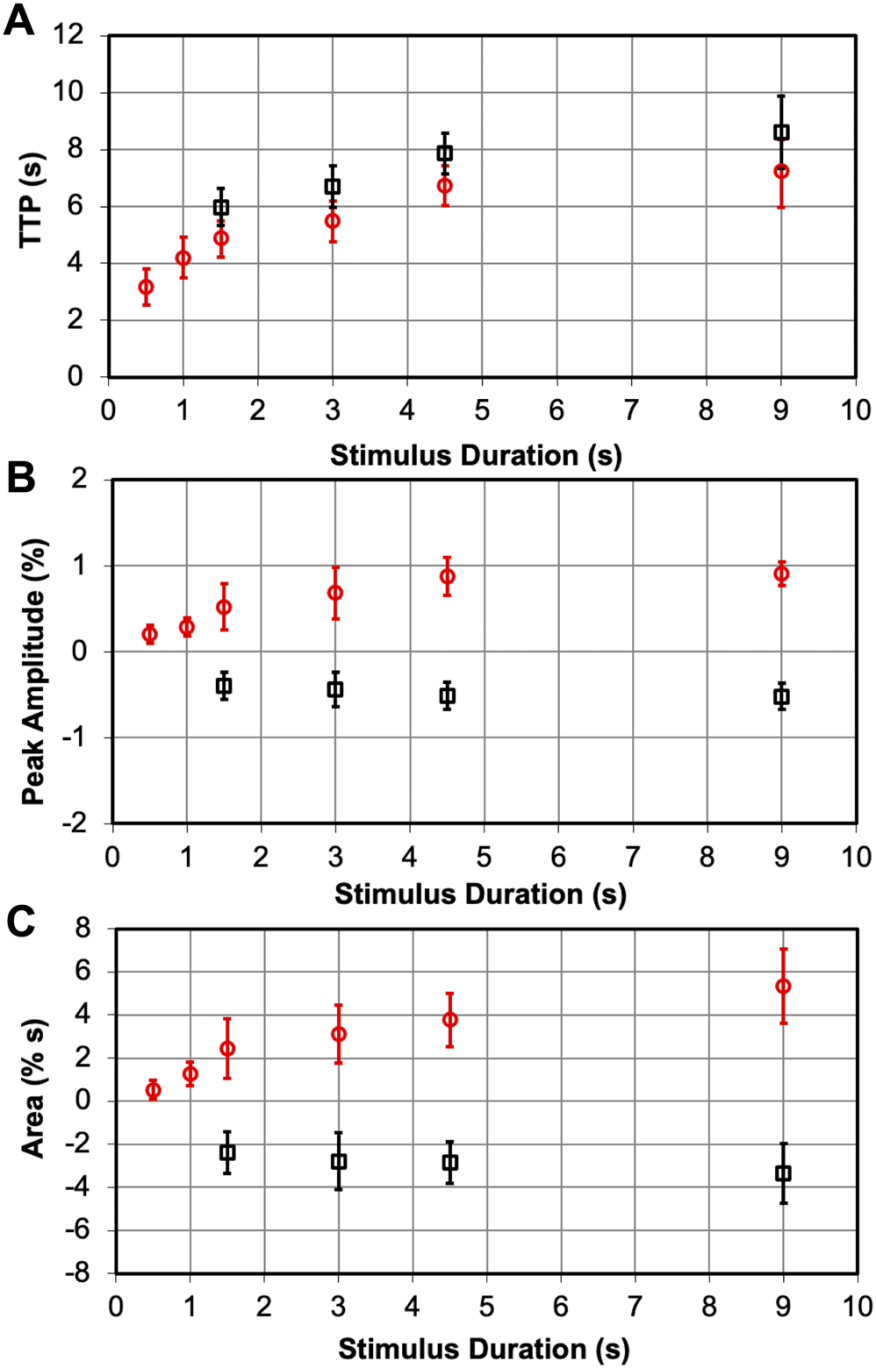
Fitting results of activation and deactivation BOLD signal at the area 3b using double gamma variate model. The obtained characteristics show their variations with changing of stimulus duration. (A) Time to peak (TTP). (B) Peak amplitude (%). (C) Area under the curve. The activations are in red, and deactivations are in black.

Quantitative comparisons (Figure 4 and Table S1) showed that TTP, peak amplitude, and area under the curve increased with stimulus duration, but NBRs consistently had longer TTP (~1.2 s), smaller amplitudes, and slower growth rates. Between 1.5 to 4.5 s, TTP, peak amplitude, and area under the curve of both PBRs and NBRs showed linear increases (but with very different slopes) with *r* ^*2*^ = 0.964, 0.997 and 0.999, and 0.983, 0.959 and 0.842, respectively (Figure 4 and Table 1). The greatest peak amplitude changes for PBRs occurred at shorter stimulus duration (0.5 to 1.5 s), which saturated at longer durations (> 4.5 s). The NBRs were weak or absent for very short durations (< 1.5 s). Moreover, the peak and amplitude had a much lower range of variation for NBRs than PBRs. In summary, while the BOLD response was well modeled by a linear dependence on the stimulus duration over some ranges, the nonlinearity in the response of these parameters at very short and long stimulus durations was also apparent. All three characteristic measures showed nonlinear relations with the stimulus duration in area 3b (Table S2), with *r* ^*2*^ larger than 0.95 for the whole stimulus duration range (0.5–9 s). Overall, PBRs and NBRs were not mirror images; their nonlinear response profiles were distinctly asymmetric.

**TABLE 1:** Results from linear fitting between BOLD characteristics and stimulus ON or OFF duration from 1.5 to 4.5 s^a^.

Response^b^	Characteristics^c^	Intercept	Slope	*r* ^2^
PBR	Area under the curve	1.768	0.447	0.999
	Peak amplitude	0.339	0.118	0.997
	Time to peak	3.835	0.623	0.964
NBR	Area under the curve	−2.223	−0.153	0.842
	Peak amplitude	−0.331	−0.039	0.959
	Time to peak	4.958	0.63	0.983

^a^ *y* = *a*_0_ + *a*_1_ *x*, *a*_0_: intercept, *a*_1_: slope. Only durations from 1.5 to 4.5 s included in fitting.

^b^ PBR, positive BOLD response (activation) for vibrotactile stimulation; NBR, negative BOLD response (deactivation) for vibrotactile stimulation interruption.

^c^ Time to peak (s), peak amplitude (%), and area under the curve (% s) were BOLD characteristics from double gamma variate model fitting of %BOLD signals in area 3b following paradigm A in Figure 1.

### 3.3 Influence of Baseline State on BOLD Response Profiles

**FIGURE 5:**
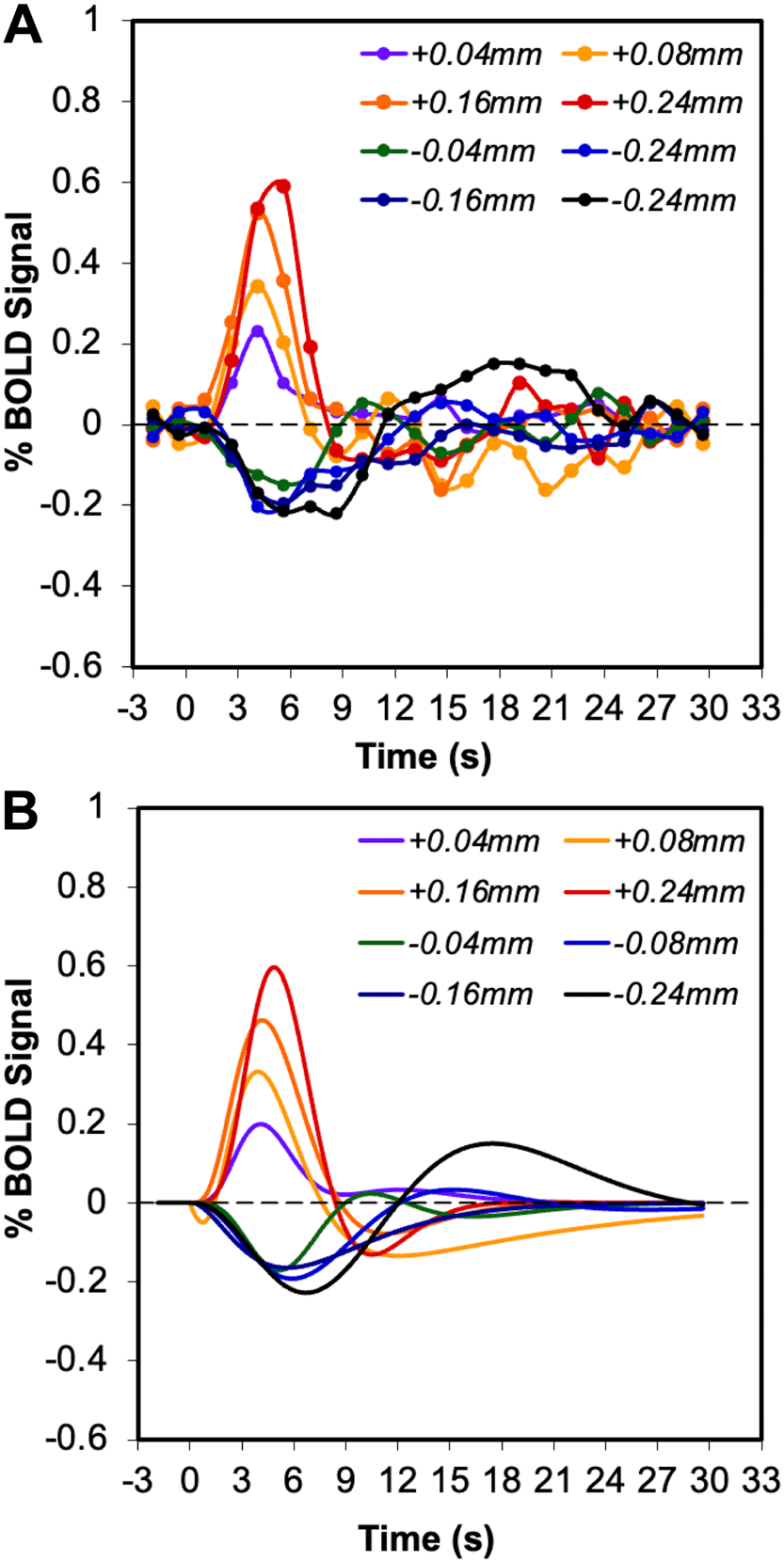
The average event‐related BOLD signal changes in area 3b in response to varying intensities of stimulus with fixed duration at 3 s. (A) Measured BOLD responses. (B) Fitted BOLD responses using the gamma‐variate function. The changes of stimulus intensity are indicated by increased (+) or decreased (−) probe displacement.

Paradigms A and B differed in baseline states so we tested where responses were symmetric about a common baseline (0.24‐mm displacement, paradigms C and D). Both PBRs and NBRs were detectable when intensity changes (±0.24 mm) lasted ≥ 1.5 s (Figure S3). At short durations, PBRs remained robust, while NBRs were weaker and more difficult to detect. With symmetric intensity changes of fixed 3‐s duration (Figure 5), PBRs again showed larger amplitudes and areas under the curve, and shorter TTPs compared with NBRs. These results confirm that asymmetry is preserved regardless of baseline condition.

### 3.4 Variation of BOLD Signals With Stimulus Intensity

**FIGURE 6:**
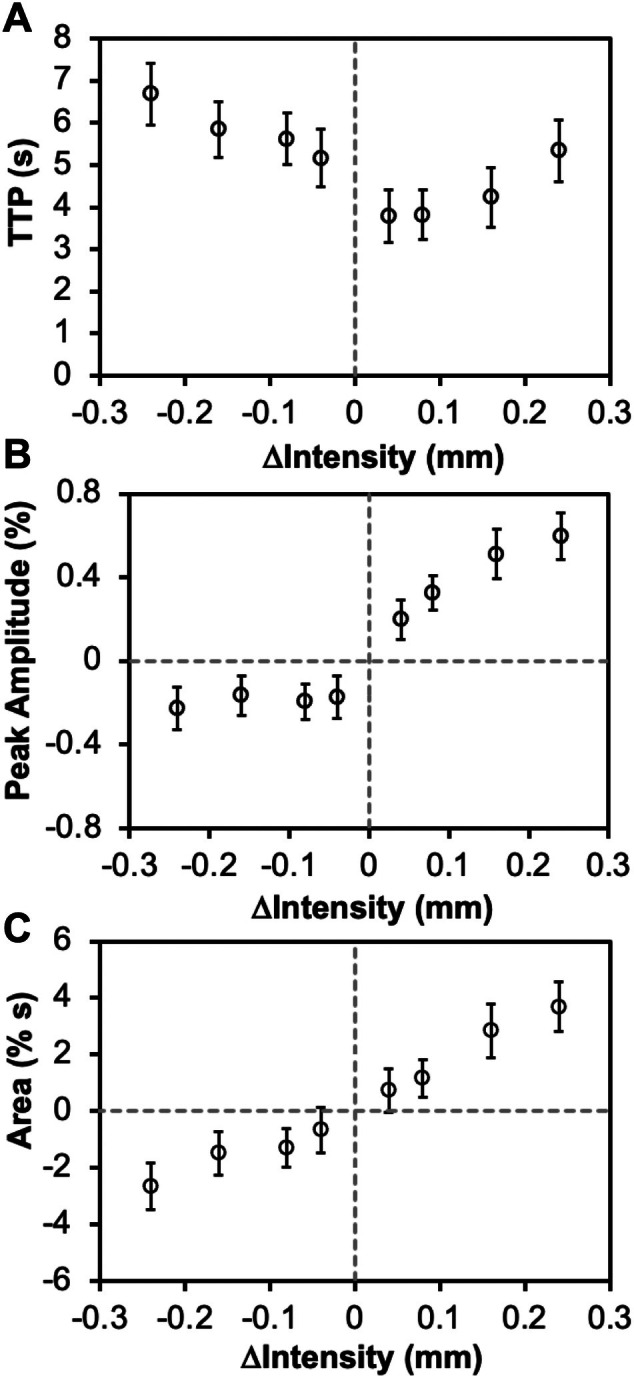
Fitting results of activation and deactivation BOLD signals in area 3b using double gamma variate model. The obtained characteristics show variations with changing of stimulus intensity indicated by probe displacement (mm). These included: (A) time to peak (TTP), (B) peak amplitude (%), and (C) area under the curve. Error bars are standard deviations across epochs. Results were from application of paradigm D shown in Figure 1.

Starting from the same active baseline (0.24 mm displacement), PBRs scaled strongly with increasing stimulus intensity (0.04–0.24 mm), showing clear linear correlations for both peak amplitude (*r* ^*2*^ = 0.960) and area under the curve (*r* ^*2*^ = 0.979) (Figures 5 and 6; Table 2) in the tested intensity range. By contrast, NBRs exhibited weaker intensity dependence: area under the curve correlated with intensity (*r* ^*2*^ = 0.917), but peak amplitude did not (*r* ^*2*^ = 0.417) and changed little over the range tested. NBRs also showed longer TTPs, slower signal decreases, and smaller amplitude changes compared with PBRs. The response pattern to increasing stimulus intensity was similar to our previous report when the stimulus was started from a neutral baseline (Zhang et al. 2007). Together, these findings demonstrate distinct nonlinear and asymmetric scaling of BOLD responses with stimulus intensity.

**TABLE 2:** Results from linear fitting between BOLD characteristics and stimulus intensity^a^.

Response^b^	Characteristics^c^	Intercept	Slope	*r* ^2^
PBR	Area under the curve	0.077	15.595	0.979
	Peak amplitude	0.150	1.989	0.960
	Time to peak	3.283	7.780	0.895
NBR	Area under the curve	−0.367	8.962	0.917
	Peak amplitude	−0.163	0.206	0.417
	Time to peak	4.923	−6.975	0.944

^a^ *y* = *a*_0_ + *a*_1_ *x*, *a*_0_: intercept, *a*_1_: slope. Stimulus intensity increased or decreased by the same amount with changes of probe displacement in the range of 0.04–0.24 mm from the same active baseline.

^b^ PBR, positive BOLD response (activation) for vibrotactile stimulation; NBR, negative BOLD response (deactivation) for vibrotactile stimulation interruption.

^c^ Time to peak (s), peak amplitude (%), and area under the curve (% s) were from double gamma variate model fitting of %BOLD signals in area 3b.

### 3.5 Correlation of MUA and BOLD Characteristics as Functions of Stimulus Intensity

**FIGURE 7:**
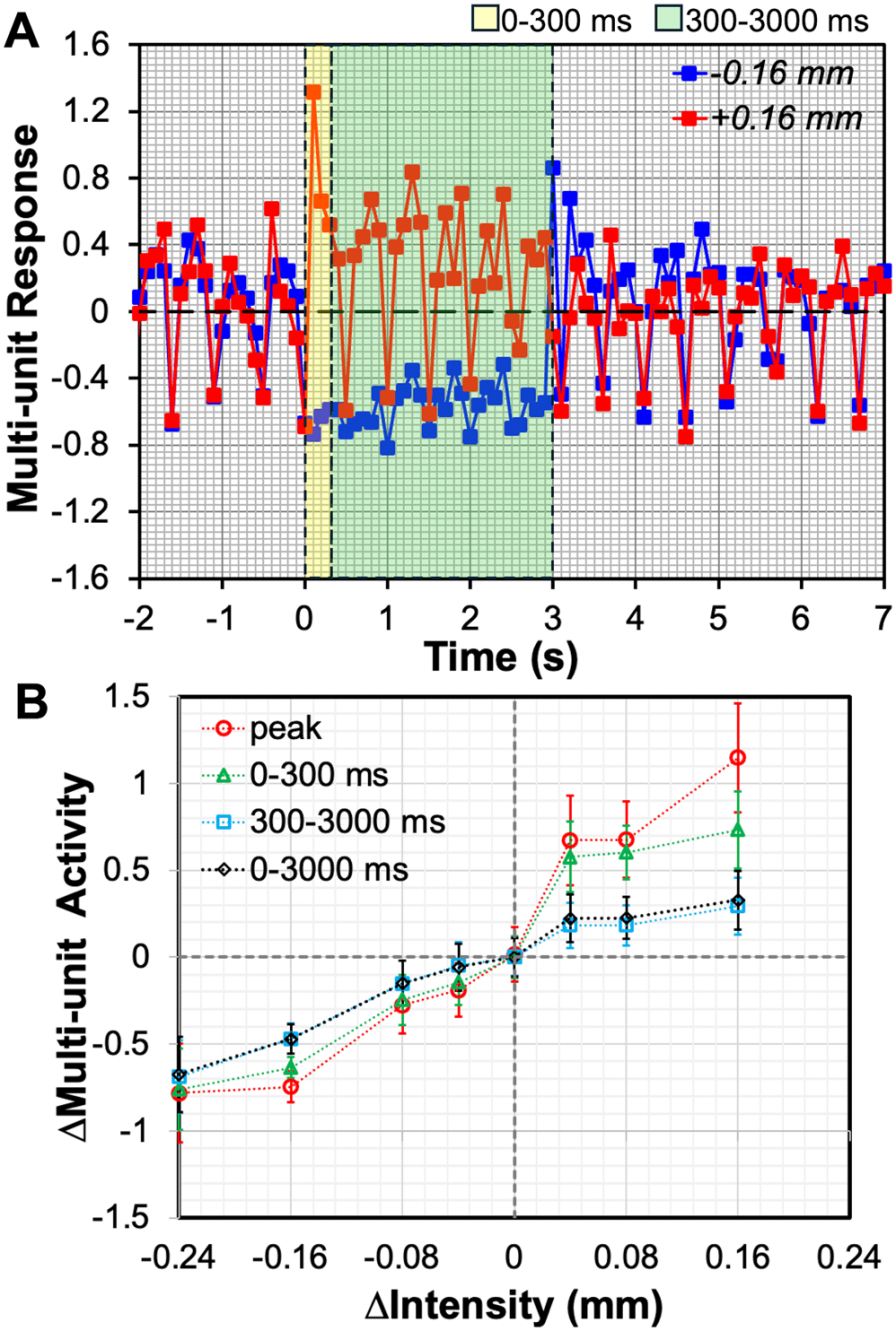
Multi‐unit activity obtained from electrophysiology. (A) Representative multi‐unit response with respect to the stimulus intensity changes (change of displacement Δ*D* at ±0.16 mm). (B) The multi‐unit activity was quantified as peak amplitude or the mean value of the activation and deactivation period (0–300, 300–3000, or 0–3000 ms).

To link BOLD signals with neural activity, we recorded multi‐unit firings in area 3b using the same stimulation paradigm (Figure 7 and Figure S1). After the initial transition phase (0–300 ms) in the stimulation baseline (0.24‐mm displacement), firing rates were stable during the sustained phase (300–4000 ms). Subsequent increases or decreases in stimulus intensity produced graded changes in firing rates, with asymmetry most pronounced during the transition phase. Increased intensity elicited rapid, transient firing bursts followed by slower sustained responses, whereas decreases produced smaller, more uniform reductions. MUAs were analyzed separately as peak amplitude, mean values of transition phase (0–300 ms), sustained phase (300–3000 ms), and all phases (0–3000 ms). Figure 7B shows the MUA changes in different phases when stimulus intensity increased or decreased from 0.24 mm active baseline by different degrees (Δ*D* at ±0.04, ±0.08, ±0.16, or −0.24 mm), respectively. The asymmetric responses to stimulus intensity change for activation and deactivation were evident.

**FIGURE 8:**
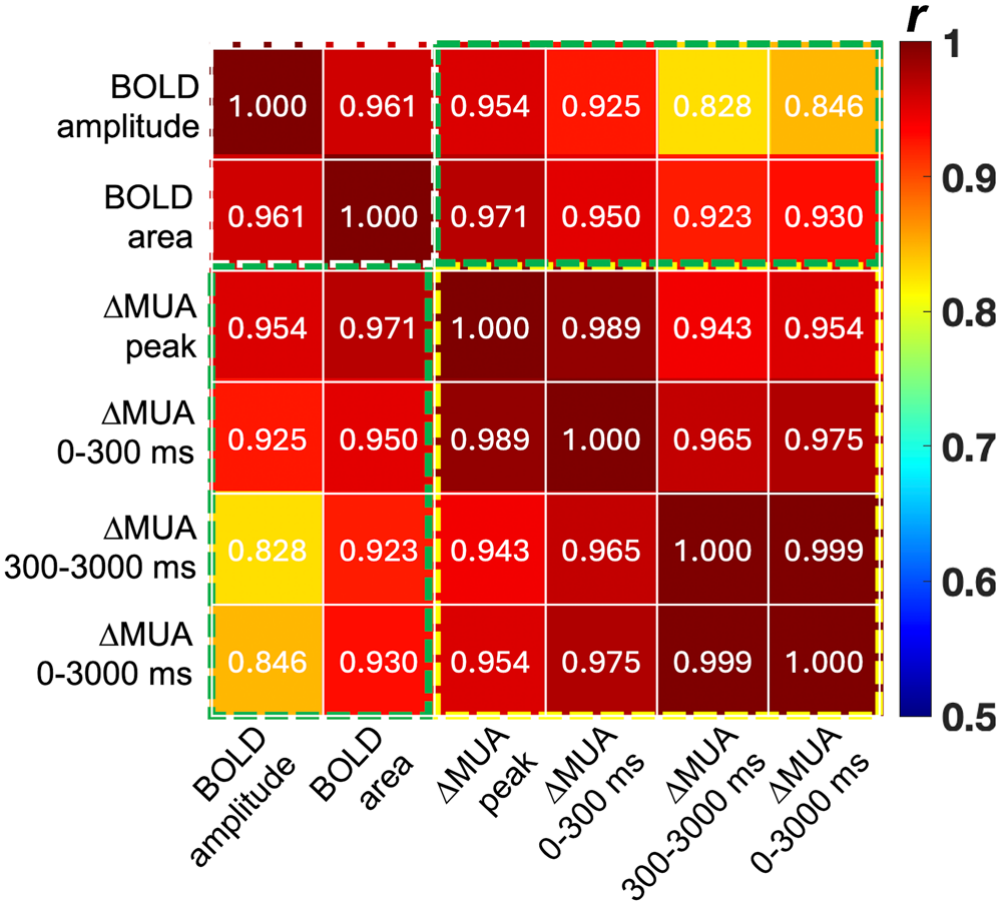
Correlation between fMRI and neural activity estimated from electrophysiology. Functional MRI BOLD characteristics (peak amplitude and area under the peak) and multi‐unit activities (MUAs) measured within the stimulated area 3b under different stimulus intensities during activation and deactivation (paradigm D in Figure 1) were compared.

Figure 8 illustrates the correlations between BOLD signal characteristics and MUA changes quantified across different phases in area 3b, including positive and negative responses corresponding to stimulus increase and stimulus reduction, respectively, from the same stimulus‐driven baseline. Quantitative analysis showed strong correlations between BOLD characteristics (peak amplitude, area under curve) and MUA changes in stimulated area 3b of primary somatosensory cortex, particularly at the peak or in the transition phase (Figure 8). Both PBRs and NBRs scaled monotonically, though at different rates, with corresponding increases or decreases in MUA. These results indicate that the nonlinear, asymmetric BOLD profiles reflect underlying differences in neural spiking during activation and deactivation.

## 4 Discussion

Our results show asymmetric positive and negative BOLD responses with nonlinear characteristics under varying stimulation conditions, including changes in duration and intensity from both resting and elevated baselines. MUAs during activation and deactivation also reflected these asymmetries.

### 4.1 Nonlinearity in Asymmetric Profiles of Positive and Negative BOLD Signals

Our results indicate that BOLD responses to changes in stimulus intensity or duration are generally nonlinear (Yesilyurt et al. 2008). They show relative insensitivity to small changes at low intensities or short durations and signal saturation at higher intensities or longer durations. Previous functional imaging studies frequently reported that positive BOLD activation maps are often accompanied by negative BOLD activations, both detected using the same HRF. However, this “mirror‐image” assumption has been challenged. In the primary visual cortex of awake humans, negative BOLD HRFs are not identical inverses of positive BOLD HRFs (Tang et al. 2009). Instead, comparable decreases in stimulus intensity evoke much weaker and slower NBRs than the PBRs produced by intensity increases, underscoring the need for distinct HRF models for negative BOLD signals (Tang et al. 2009).

This study extends previous work by examining whether similar asymmetries exist in the somatosensory cortex (area 3b) of anesthetized New World monkeys. The results confirm that negative BOLD signals arise from an HRF that is qualitatively similar but quantitatively different from the HRF for positive responses. This asymmetry represents another form of nonlinearity, in addition to the failure of linear superposition in predicting responses to prolonged or stronger stimuli. In general, equal magnitudes of stimulus increases and decreases evoked greater positive than negative BOLD signals. NBRs rose more slowly (longer TTP), with smaller amplitude and area. MUA recordings under the same paradigm revealed asymmetric activation and deactivation profiles (Figure 7), especially during transitions. This may relate to firing rate changes in subsets of area 3b neurons, such as rapidly adapting neurons. After a period of high‐frequency firing, these neurons exhibit a decrease in their firing rates even though the excitatory input remains constant, as a form of intrinsic regulation that prevents over‐excitation. Faster firing increases during transitions stimulate greater blood flow through neurovascular coupling, enabling rapid oxygen delivery, possibly explaining the faster TTP and stronger PBRs compared to NBRs. These results suggest that different mathematical models are needed for predicting positive versus negative BOLD responses.

We used a standard GLM and HRF to detect the BOLD signal changes. This choice could bias the detection since a model‐free approach was not attempted in the current study. However, subsequent measures of percent signal changes and epoch‐averaged time courses did not assume the same HRF. The choice of two voxels with the lowest *p*‐values within area 3b for BOLD response analysis was based on two primary considerations. First, we expect BOLD signal increases to be spatially confined to the digit‐associated representation within area 3b. Previous mapping studies of hand and digit representation in area 3b of squirrel monkeys have shown that the cortical territory corresponding to a single digit or two adjacent digits is approximately 0.8 mm^2^ (Chen et al. 2003; Shi et al. 2019, 2017). Second, the spatial resolution of our EPI resolution was 0.625 × 0.625 mm^2^, such that the digit representation corresponds to approximately two EPI voxels. Selecting the two most significantly activated voxels therefore optimizes the SNR of the BOLD signal, reduces partial volume effects, and allows for comparison with electrophysiological results (Shi et al. 2019).

### 4.2 Interpretation of BOLD Characteristics From Nonlinearity of MUA Responses

The asymmetry between PBRs and NBRs likely reflects asymmetrical neuronal firing rates. MUA generally increases with stimulation intensity in a nonlinear fashion, reflecting how neuronal populations encode stimulus magnitude. This response depends on both the firing rates of individual neurons and the recruitment of additional neurons at higher intensities (Muniak et al. 2007). Neural encoding of stimulus intensity is not a simple one‐to‐one relationship. Several mechanisms shape MUA responses, including rate coding, population coding, nonlinear firing, response probability, and modulation of population dynamics (Glazewski and Barth 2015; Muniak et al. 2007). In rate coding, a neuron's firing rate scales with stimulus intensity: weak stimuli produce slow, irregular firing, whereas stronger stimuli elicit faster, more regular firing. Recruitment adds further complexity, as higher‐intensity stimuli activate additional neurons. For example, a light touch activates few sensory neurons, while a strong pinch recruits many more. As a result, population responses often saturate at high intensities, with linearity only present in a mid‐range. At low stimulus intensities, the probability of neuronal firing decreases, reducing the fraction of responsive neurons despite ongoing activity in some cells.

Multi‐unit spiking recordings in this study revealed nonlinear responses, including asymmetric activation and deactivation profiles, dynamic time‐domain changes, and nonlinear scaling with stimulus intensity. Under identical paradigms, MUA correlated strongly with BOLD characteristics across intensities. The strong correlation suggests that the same mechanisms driving MUA nonlinearities may also govern BOLD responses and arise from neural activities rather than vascular effects. Importantly, firing rates and population dynamics scale differently during activation versus deactivation, with more evident asymmetry observed in the early transition than in the later sustained phase. The highest correlations between BOLD responses and the peak/transition phase of ΔMUA suggest that HRF asymmetry may mainly arise from early ΔMUA dynamics. Nevertheless, this correlational analysis has limitations. BOLD and MUA signals were recorded in separate sessions, so simultaneous BOLD and MUA recording studies are necessary to conclude that neural activity contributes to the temporal and intensity‐dependent response features of positive and negative BOLD responses. Together, the findings presented here suggest that nonlinear MUA activity may underlie the asymmetric and nonlinear features of positive and negative BOLD responses.

### 4.3 Effects of Anesthetics

It is important to note that anesthesia can influence fMRI observations, particularly when results are directly related or compared with BOLD signals acquired in awake subjects. In general, anesthetized animals show weaker functional responses to stimulation. To ensure detectable functional responses, the anesthesia level was carefully monitored and maintained during our studies, including heart rate, SpO_2_, and ET‐CO_2_. The previous studies (Ann Stringer et al. 2014) have reported that non‐anesthetized humans showed quite similar focal responses to vibro‐tactile stimulations in area 3b, but the percentage signal change was much stronger (~4 times) compared to what was observed in anesthetized squirrel monkeys. Based on prior literature (Sellers et al. 2015) and our previous studies (Wu et al. 2016), the commonly reported effects of general anesthesia (such as isoflurane used in the present study) include: (1) a reduction in amplitude or duration of stimulus‐evoked responses compared with lighter anesthesia or awake conditions; (2) decreased resting‐state functional connectivity, as measured by temporal synchrony; (3) alterations in the temporal dynamics of responses; and (4) modified interactions between cortical areas. In the current study, the effects of anesthesia are likely reflected in reduced BOLD and MUA response amplitudes under stimulation conditions. Nevertheless, we reliably detected differences across varying intensities and durations of tactile stimulation. We attribute this robustness to the improved BOLD signal‐to‐noise ratio at high‐field (9.4 T) MRI. As we have shown previously, although anesthesia suppresses the amplitude of stimulus‐evoked BOLD responses, it also reduces noise due to carefully maintained physiological conditions and controlled ventilation (Chen et al. 2005). While dynamic features of neuronal signals may be more strongly affected by anesthesia, we did not specifically characterize the temporal features of the negative BOLD signal changes in the present study.

Importantly, the primary focus of the current study is on amplitude‐based changes across stimulus intensities and durations rather than absolute response magnitude or temporal dynamics. Thus, while anesthesia is expected to suppress overall response amplitudes, there is currently no strong evidence that it fundamentally alters the stimulus dose–response relationship for BOLD signals. The relative scaling of responses with stimulus parameters is expected to be preserved. Therefore, we believe our findings remain informative for understanding BOLD responses at the level examined here. That said, we acknowledge that anesthesia may differentially affect specific aspects of cortical processing, including laminar‐specific responses and high‐frequency neural activity.

### 4.4 Difference Between Vascular‐Tone‐Dependent and Inhibition‐Associated NBRs

There is both an important conceptual and mechanistic distinction between the negative BOLD response (NBR) in the stimulated region examined in the current study and the more commonly reported inhibition‐associated NBR in non‐stimulated regions observed below a resting baseline. In the present study, the interruption‐induced signal decrease reflects a reduction to a stimulus‐driven constant activation baseline, resulting from the withdrawal of ongoing excitatory input. This differs fundamentally from some other NBR paradigms, in which the BOLD signal decreases below the resting baseline in non‐stimulated regions and it is thought to arise, at least in part, from active neural inhibition and associated reductions in CBF and oxygen metabolism (Shmuel et al. 2006, 2002).

The primary goal of the present study was to characterize how BOLD signals behave when the baseline cortical state itself is systematically elevated by sustained stimulation with varying intensity. As discussed, this sustained active cortical state represents a fundamentally different physiological and vascular condition compared with the resting baseline, with altered vascular tone, baseline CBF, and neurovascular coupling compared with resting conditions. Under such circumstances, stimulus interruption probes the dynamics of neurovascular responses around an already vasodilated baseline, rather than invoking inhibitory mechanisms that suppress activity below rest.

## 5 Conclusion

Our results show asymmetric positive and negative BOLD responses for activation and deactivation, and nonlinear BOLD characteristics to various stimulation conditions including variations in duration and intensity from both resting and elevated baselines. BOLD characteristics have been highly associated with neural multi‐unit firing properties, revealing the asymmetry and nonlinearity of neural responses observed in activation and deactivation.

## Funding

This work was supported by the National Institutes of Health (NINDS‐2R01NS078680).

## Supporting information


**Table S1:** Characteristics of positive and negative BOLD response profiles at different stimulus ON or OFF duration^a^.
**Table S2:** Results for the 3rd order polynomial fitting between BOLD characteristic and the stimulus duration^a^.
**Table S3:** Results from the linear regression between BOLD characteristic and the mid‐level stimulus intensity^a^.
**Figure S1:** Quantification of the normalized firing counts from electrophysiological experiments. (A) The time courses of average firing counts across 9 experiments following the stimulation paradigms shown on the top. The activation and deactivation started from a stimulation baseline (probe displacement △*D*_0_ = 0.24 mm). Stimulus intensity increases or decreases by different scale, indicated by further change of displacement △*D* with the same stimulus duration at 3 s. The early period (0–300 ms) shows much larger alteration than the sustained period (300–3000 ms). (B) The time courses of average counts across experiments (*N* = 9) after normalization based on the mean of the stimulation baseline of each experiment. The normalization was done to reduce the difference in stimulation baseline, which might affect the assessment of neural response to further activation and deactivation starting from the stimulation baseline. Due to the evident difference in early and late activation or deactivation period, the multiunit activity was further quantified for periods 0–300, 300–3000, and 0–3000 ms during activation or deactivation obtained from electrophysiology.
**Figure S2:** The average event‐related BOLD signal Changes in the *area 3b* in the somatosensory cortex in response to varying durations of stimulus interruption. Measured BOLD responses (indicated by markers) and fitted BOLD responses (indicated by lines) using the gamma‐variate function.
**Figure S3:** The average BOLD signal changes in area 3b in response to varying stimulus duration. The markers indicate the measured BOLD responses, while the lines indicate the fitted BOLD responses using the gamma‐variate function. The stimulus intensity increased or decreased by 0.24 mm probe displacement, starting from the same active baseline of 0.24 mm displacement (paradigm C in Figure 1). The activation and deactivation durations altered (1.5, 3.0, and 9.0 s). The filled markers for activation and blank markers for deactivation.

## Data Availability

The data that support the findings of this study are available from the corresponding author upon reasonable request.
