# Supplementary material for: Nonlinear Asymmetric Blood Oxygenation Level Dependent Responses in Somatosensory Cortex
Source: Hum Brain Mapp. 2026 Apr 7;47(5):e70523. doi: 10.1002/hbm.70523 (PMC13057424; doi:10.1002/hbm.70523)
Supplement: Supplementary file 1 — Table S1: Characteristics of positive and negative BOLD response profiles at different stimulus ON or OFF durationa. Table S2: Results for the 3rd order polynomial fitting between BOLD characteristic and the stimulus durationa. Table S3: Results from the linear regression between BOLD characteristic and the mid‐level stimulus intensitya. Figure S1: Quantification of the normalized firing counts from electrophysiological experiments. (A) The time courses of average firing counts across 9 experiments following the stimulation paradigms shown on the top. The activation and deactivation started from a stimulation baseline (probe displacement △D 0 = 0.24 mm). Stimulus intensity increases or decreases by different scale, indicated by further change of displacement △D with the same stimulus duration at 3 s. The early period (0–300 ms) shows much larger alteration than the sustained period (300–3000 ms). (B) The time courses of average counts across experiments (N = 9) after normalization based on the mean of the stimulation baseline of each experiment. The normalization was done to reduce the difference in stimulation baseline, which might affect the assessment of neural response to further activation and deactivation starting from the stimulation baseline. Due to the evident difference in early and late activation or deactivation period, the multiunit activity was further quantified for periods 0–300, 300–3000, and 0–3000 ms during activation or deactivation obtained from electrophysiology. Figure S2: The average event‐related BOLD signal Changes in the area 3b in the somatosensory cortex in response to varying durations of stimulus interruption. Measured BOLD responses (indicated by markers) and fitted BOLD responses (indicated by lines) using the gamma‐variate function. Figure S3: The average BOLD signal changes in area 3b in response to varying stimulus duration. The markers indicate the measured BOLD responses, while the lines indicate the fitted BOLD responses using [file HBM-47-e70523-s001.docx]

**Supporting Information**

**Supporting Table S1**. Characteristics of positive and negative BOLD response profiles at different stimulus ON or OFF duration.^a^

| **Duration**  **(s)** | **Time to peak (s)** | | **Peak amplitude (%)** | | **Area (% s)** | | **FWHM (s)** | |
| --- | --- | --- | --- | --- | --- | --- | --- | --- |
|  | **PBR** | **NBR** | **PBR** | **NBR** | **PBR** | **NBR** | **PBR** | **NBR** |
| 0.5 | 3.19 |  | 0.21 |  | 0.53 |  | 2.98 |  |
| 1.0 | 4.19 |  | 0.29 |  | 1.27 |  | 4.87 |  |
| 1.5 | 4.88 | 5.98 | 0.52 | -0.40 | 2.43 | -2.40 | 3.46 | 5.82 |
| 3.0 | 5.50 | 6.71 | 0.68 | -0.43 | 3.13 | -2.80 | 4.27 | 6.13 |
| 4.5 | 6.75 | 7.87 | 0.88 | -0.51 | 3.77 | -2.85 | 4.55 | 6.54 |
| 9.0 | 7.23 | 8.61 | 0.91 | -0.52 | 5.34 | -3.37 | 10.77 | 10.53 |
| 30.0 | 12.38 | 13.59 | 0.93 | -0.54 | 30.75 | -18.47 | 33.37 | 33.97 |

^a^Fitting results of %BOLD signals in area 3b using double gamma variate model.

The obtained characteristics included: time to peak (TTP), peak amplitude (%), and area under the curve (% Sec). PBR, positive BOLD response (activation) for vibrotactile stimulation shown as conventional paradigm A in Figure 1; NBR, negative BOLD response (deactivation) for vibrotactile stimulation interruption shown as reversed paradigm B in Figure 1.

**Supporting Table S2**. Results for the 3^rd^ order polynomial fitting between BOLD characteristic and the stimulus duration.^a^

| **Response^b^** | **Characteristics^c^** | **a_0_** | **a_1_** | **a_2_** | **a_3_** | **r^2^** |
| --- | --- | --- | --- | --- | --- | --- |
| **PBR** | Area under the curve | 0.205 | 1.263 | 0.107 | 0.003 | 0.999 |
|  | Peak amplitude | 0.073 | 0.291 | 0.028 | 0.006 | 0.984 |
|  | Time to peak | 2.801 | 0.334 | 0.119 | 0.003 | 0.994 |
| **NBR** | Area under the curve | -2.057 | -0.284 | 0.026 | -0.001 | 1.000 |
|  | Peak amplitude | -0.296 | -0.071 | 0.007 | -0.000 | 0.952 |
|  | Time to peak | 4.505 | 1.040 | -0.082 | 0.002 | 0.998 |

^a^y=a_0_+a_1_x+a_2_x^2^+a_3_x^3^

**^b^**PBR, positive BOLD response (activation) for vibrotactile stimulation; NBR, negative BOLD response (deactivation) for vibrotactile stimulation interruption.

**^c^**Time to peak (TTP), peak amplitude (%), and area under the curve (% Sec) were fitting results of %BOLD signals in area 3b using double gamma variate model.

**Supporting Table S3**. Results from the linear regression between BOLD characteristic and the mid-level stimulus intensity.^a^

| **Response^b^** | **Characteristics^c^** | **Intercept** | **Slope** | **r^2^** |
| --- | --- | --- | --- | --- |
| **PBR** | Area under the curve | -3.763 | 31.639 | 0.964 |
|  | Peak amplitude | -0.206 | 3.419 | 0.957 |
|  | Time to peak | 0.640 | 19.125 | 0.937 |
| **NBR** | Area under the curve | 1.555 | -16.875 | 0.848 |
|  | Peak amplitude | -0.118 | -0.438 | 0.787 |
|  | Time to peak | 3.402 | 13.250 | 0.898 |

**^a^**y=a_0_+a_1_x, a_0_: intercept, a_1_: slope. Only the mid-level stimulus intensities (displacement from 0.16 mm to 0.24 mm) were included in fitting.

^b^PBR, positive BOLD response (activation) for vibrotactile stimulation; NBR, negative BOLD response (deactivation) for vibrotactile stimulation interruption.

**^c^**Time to peak (s), peak amplitude (%), and area under the curve (% s) were fitting results of %BOLD signals in area 3b using double gamma variate model.


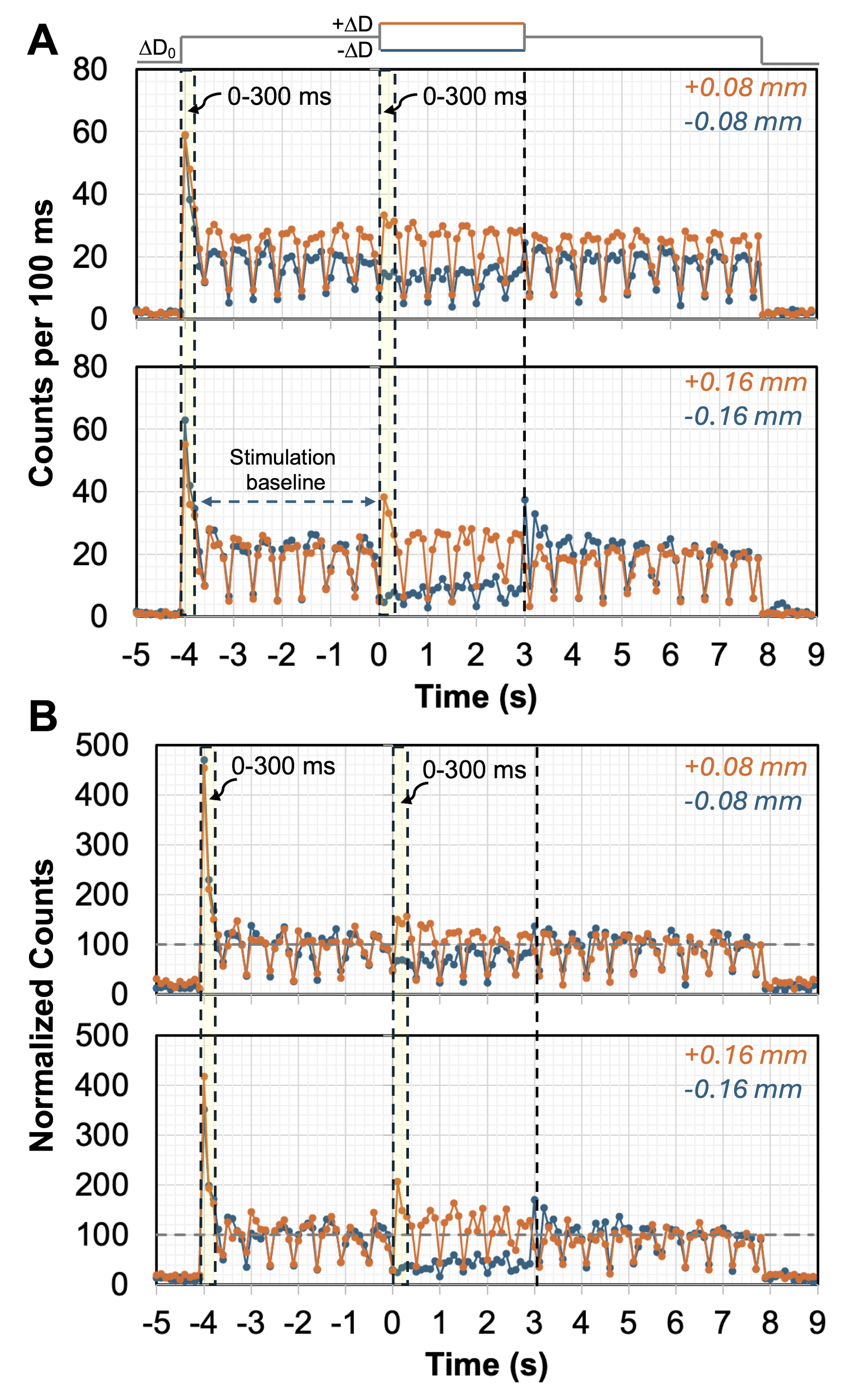


**Supporting Figure S1**. Quantification of the normalized firing counts from electrophysiological experiments. (A) The time courses of average firing counts across 9 experiments following the stimulation paradigms shown on the top. The activation and deactivation started from a stimulation baseline (probe displacement ΔD_0_ = 0.24 mm). Stimulus intensity increases or decreases by different scale, indicated by further change of displacement ΔD with the same stimulus duration at 3s. The early period (0-300 ms) shows much larger alteration than the sustained period (300-3000 ms). (B) The time courses of average counts across experiments (N = 9) after normalization based on the mean of the stimulation baseline of each experiment. The normalization was done to reduce the difference in stimulation baseline, which might affect the assessment of neural response to further activation and deactivation starting from the stimulation baseline. Due to the evident difference in early and late activation or deactivation period, the multiunit activity was further quantified for periods 0-300, 300-3000, and 0-3000 ms during activation or deactivation obtained from electrophysiology.


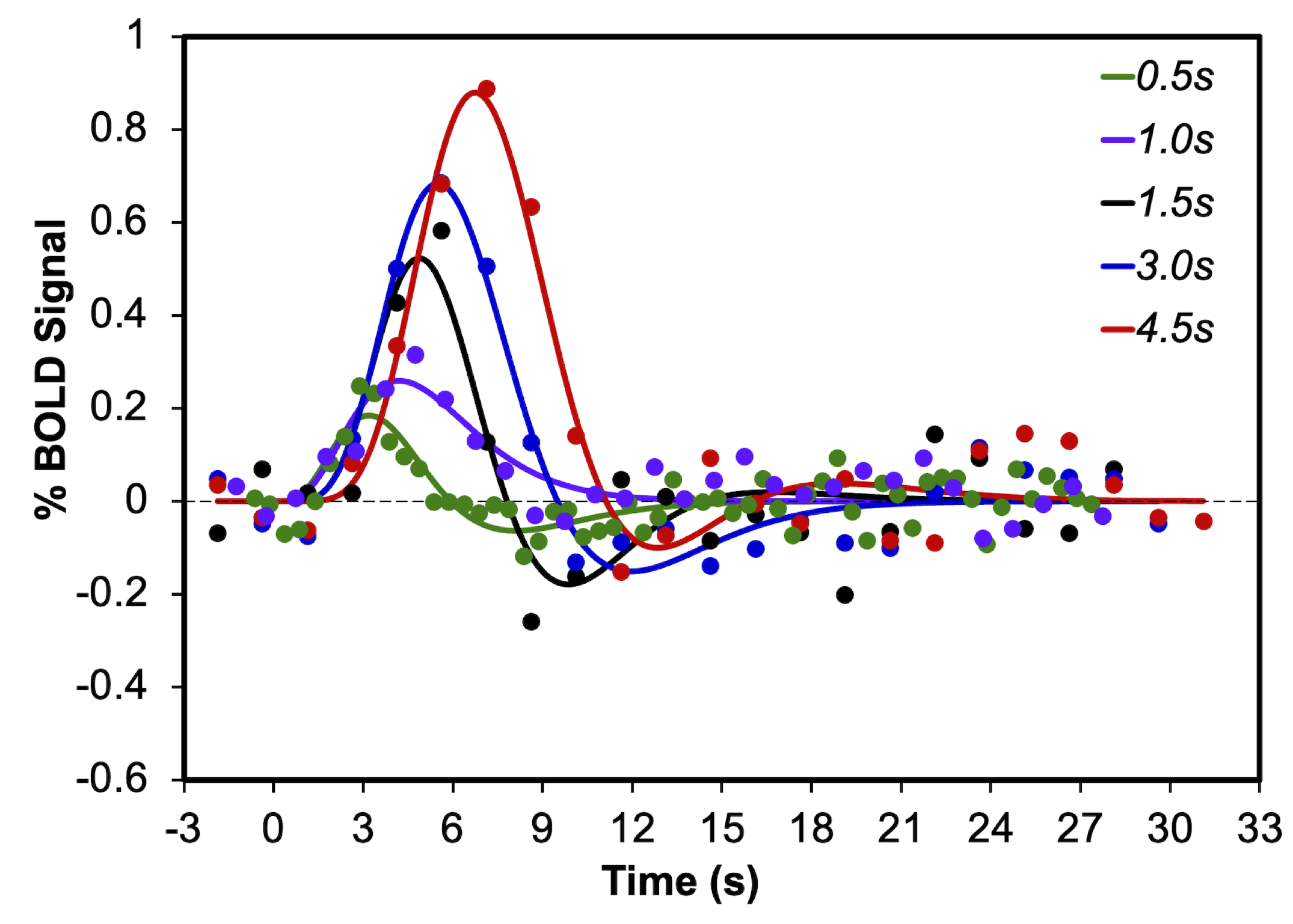


**Supporting Figure S2**. The average event-related BOLD signal Changes in the *area 3b* in the somatosensory cortex in response to varying durations of stimulus interruption. Measured BOLD responses (indicated by markers) and fitted BOLD responses (indicated by lines) using the gamma-variate function.


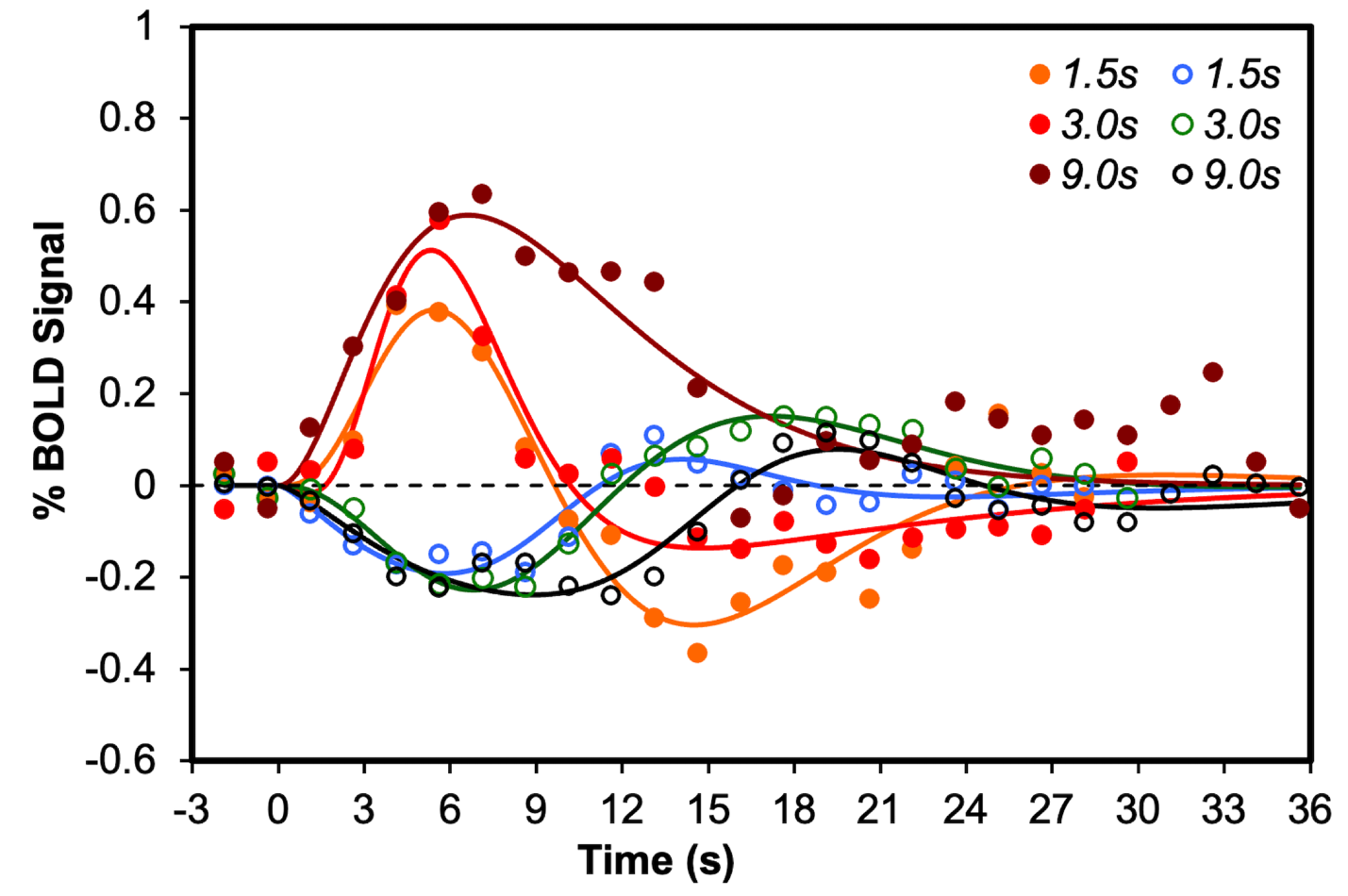


**Supporting Figure S3**. The average BOLD signal changes in area 3b in response to varying stimulus duration. The markers indicate the measured BOLD responses, while the lines indicate the fitted BOLD responses using the gamma-variate function. The stimulus intensity increased or decreased by 0.24 mm probe displacement, starting from the same active baseline of 0.24 mm displacement (paradigm C in Figure 1). The activation and deactivation durations altered (1.5, 3.0, and 9.0 s). The filled markers for activation and blank markers for deactivation.
